# Changing Course: An Educational Tool for Antipsychotic Switching

**DOI:** 10.1192/bjo.2025.10294

**Published:** 2025-06-20

**Authors:** Alexander Noar

**Affiliations:** North London Foundation NHS Trust, London, United Kingdom. University College London, London, United Kingdom

## Abstract

**Aims:** There is a notable lack of standardised guidelines on antipsychotic switching, including in the Maudsley Prescribing Guidelines. This presents a challenge for psychiatrists who must navigate complex decisions regarding efficacy, side effects, and receptor-binding properties when transitioning patients between antipsychotics. This project aimed to develop an educational tool that synthesizes information on antipsychotic efficacy and receptor profiles to assist clinicians in making evidence-based switching decisions. The tool was inspired by the need for a structured approach to antipsychotic transitions, incorporating data from Stahl’s Essential Psychopharmacology and relevant receptor-binding research.

**Methods:** A comprehensive literature review was conducted to consolidate information on the pharmacodynamics and efficacy of commonly used antipsychotics. Research studies detailing receptor affinities for dopamine, serotonin, histamine, muscarinic, and adrenergic receptors were examined.

**Results:** The educational tool provides a structured framework for psychiatrists, offering guidance on selecting an appropriate switching strategy. The educational tool was designed to visually present this information, allowing clinicians to compare medications based on receptor binding, side effect profiles, and equivalent dosing strategies. It also included switching strategies, emphasizing cross-titration and pharmacodynamic considerations to minimize withdrawal and receptor rebound effects. It highlights receptor-mediated rebound effects e.g., H1-related insomnia, M1-related agitation. By integrating receptor-based pharmacological data with practical clinical considerations, the tool enhances decision-making in scenarios where guidelines are lacking.

**Conclusion:** The absence of clear guidelines for antipsychotic switching necessitates a standardized, evidence-based approach. This educational tool consolidates pharmacological knowledge to aid psychiatrists in optimizing treatment transitions, minimizing withdrawal effects, and improving clinical outcomes. Future iterations could incorporate real-world validation studies to assess its impact on prescribing decisions and patient outcomes.

